# A Study on the Dimension Accuracy on the Inner Structure of the 3D Printed Parts Caused by the Scanning Strategy

**DOI:** 10.3390/ma12081333

**Published:** 2019-04-24

**Authors:** Jitai Han, Meiping Wu, Yanan Ge

**Affiliations:** 1 School of Mechanical Engineering, Jiangnan University, Wuxi 214122, China; wmp116699@163.com (M.W.); 18851501880@163.com (Y.G.); 2 Additive Manufacturing Products Supervision and Inspection Center of Jiangsu Province, Wuxi Institution of Supervision & Testing on Product Quality, Wuxi 214073, China

**Keywords:** selective laser melting (SLM), dimension accuracy, inner structure, scanning strategy, Ti6Al4V

## Abstract

Selective laser melting (SLM) has been used in many fields recently, especially in the aerospace field. Many studies have been done on mechanical properties of the printed parts, but the dimension accuracy of the inner structure received little attention during these years. In this work, the dimension accuracy of the inner structure was measured and compared using different scanning strategies. Compared with the measured data, a new scanning strategy was used and finds that the dimension accuracy was better than the previous one that used a two-scanning strategy. To explain this phenomenon, finite element analysis (FEA) was used to show the temperature distribution after a 0.1 s cooling using two different scanning strategies, which caused the dimensional deviation in printing.

## 1. Introduction

Additive manufacturing (AM), which is known as three-dimensional printing, has drawn increasing attention in recent years. The complete manufacturing process begins in a computer. The designer draws a 3D computer aided design (CAD) model on the computer and imports this file to the software of the three dimensional printer. Then the 3D CAD file is sliced into layers in the software and converts into an STL format file. The software delivers the relative information about layers to the 3D printer and controls the laser melting the power on the specific area accordingly. When one layer finishes, the work plate moves down by one layer height and the powder paves the layer by the feeder system. This whole process will be repeated until printing out the product. 

From the illustration of the printing process, it can be easily found that AM has quite a lot of advantages compared to a traditional manufacturing method. First, it is easier to control. Methods like milling, grinding, and planning are no longer needed. With a computer and 3D printer, products can be built directly. Secondly, it is much easier to increase the mechanical property of the printed parts by optimizing the component of the raw material. Third, it saves quite a lot of money since it wastes much less material compared to the traditional methods. Fourth, 3D printing makes it possible to print objects with a complex internal structure and some other advantages, such as being lightweight, saving time, and so on, which are not listed here. Many studies on this technology have been done in recent years. Di Wang et al. [1] fabricated overhanging structures with different inclined angles and designed the thin walls and cylinders with different geometrical dimensions to get the critical inclined angle for designing the porous structure and optimize the fabricating resolution. Zhe Yang et al. [2] fabricated a 316L stainless steel thin-walled circular tube with preset internal circumferential rectangular groove defects using the SLM method. Johannes Günther et al. [3] assessed the fatigue properties of Ti-6Al-4V samples designed with internal axial channels featuring a rough as-built surface. Relatively low scatter of fatigue lives found is attributed to rapid crack initiation and, thus, the dominant influence of the micro-crack growth regime. Many researchers are now focusing on this technology in different kinds of fields, especially in the aerospace industry. Miguel Seabra et al. [4] defined a design methodology of aircraft bracket topology in order to facilitate and make the topology optimization solution design more accurate and make it ready for AM. Blades with less weight and better aerodynamic property are always needed, but cannot be achieved by traditional manufacturing methods. However, with the help of 3D printing, this requirement can be satisfied. Although printing a blade with an internal structure is not difficult, it is still difficult to print an object with an accurate dimension and geometric tolerance of the internal structure to meet the need of aerodynamics. 

Some works have been done to improve the accuracy of the printed sample. Zaeh and Branner [5] found that the thermal gradient along with the related plastic behavior will likely lead to a reduction in dimensional accuracy as well as an increase in the residual stress and thermally-induced cracks in a dimensional part. Bremen, Meiners, and Diatlov [6] showed that a larger diameter beam could also cause a large melt pool, which leads to a lower dimensional accuracy. However, a problem Bremen wrote in this paper is about circumventing using a laser source with variable focus diameters. Prem et al. [7] showed that both the scanning speed and laser power can affect the dimensional accuracy of the printed parts. In fact, it can also strongly affect the dimension on a different axis. Furthermore, Jitai et al. [8] found that the surface quality is highly affected by the scanning strategies. In fact, some simulations are also done on the residual stresses and distortion of the printed parts, which can highly affect the dimensional accuracy of the printed parts. Kruth et al. [9] prepared a novel analysis method to compare the influence of the processing parameters on residual stresses during printing. Hodge, Ferencz, and Solberg [10] used a thermomechanical model to simulate the complete printing process and found better performance of the simulation results. Li et al. [11,12] did three relative research studies on the prediction model in selective laser melting and found that the multiscale modeling approach was an effective way for fast prediction of the part distortion. Zhuang et al. [13] found that the RSM technique in conjunction with the process window to filter those unsuitable processing parameters could be a more effective way to achieve good accuracy of the predictions for melt pool dimensions during SLM. Radek Vrána et al. [14] printed four types of specimens from AlSi10Mg alloy powder material and found that, in the case of an elliptical cross-section, a significantly better match was found (2% error in the F_max_) with the low-velocity impact experiments during the whole deformation process compared with the circular cross-section. Another work [15] discussed the influence of boundary conditions during LBM of EN AW-2219 on sample porosity and found that elongation at the break of T6 specimens loaded along the build-up direction exceeded the values from literature for conventionally manufactured EN AW-2219.

From the introduction given above, we can find that, although some research studies have been done on the accuracy of the printed parts and inner structure. However, it draws little attention in recent work. In this work, finite element analysis was used to illustrate this phenomenon in theory and a modified scanning strategy was used to print a specimen with more accurate internal structures compared to the previous scanning strategies.

## 2. Experiments

Plasma Rotating Electrode Process (PREP) Ti-6Al-4V powder supplied by SHENZHEN MINATECH CO., LTD., Shenzhen, China, was used in this study. The chemical compositions and other parameters are listed in Table 1. The printer used in this research was FS271M, Farsoon, Changsha, China and the engineers optimized all the printing parameters. The printing parameters were maintained the same during the entire printing process except the scanning strategy. The program preset in the software including the following three strategies shown in Figure 2 changed the scanning strategy. Other relative processing parameters can be found in Table 2.

**Figure 1:**
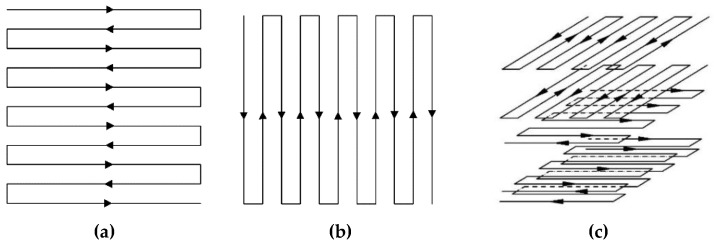
Three different zigzag strategies. (**a**) x-axis scanning strategy (**b**) y-axis scanning strategy, (**c**) multi-direction scanning strategy.

**Figure 2:**
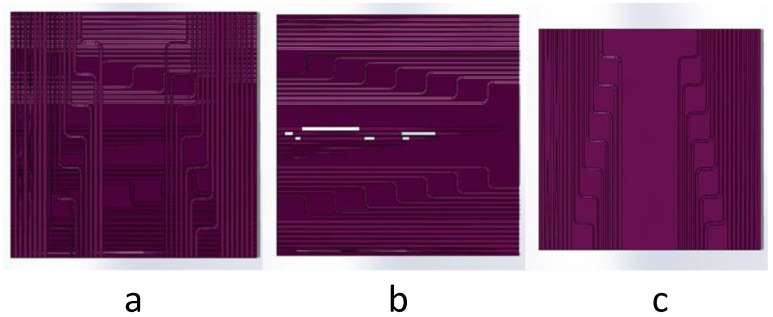
Whole design, the first part of the specimen, and the second part of the specimen shown in (**a**–**c**) separately.

The scanning strategy used in this research was shown in Figure 2. The first scanning strategy was along x-axis and the second scanning strategy was along the y-axis listed in Figure 1a,b, separately. As for the last scanning strategy, it was a mixture of the previously mentioned scanning strategy. This scanning strategy was modified according to both the thickness of the specimen and one layer thickness. The first part was printing along the x-axis and the second part was printing along y-axis, which is shown in Figure 1c. 

Due to the lack of the design drawing to study the inner structure, a new model was designed with different kinds of structural features, as shown in Figure 2. The features on the first part (shown in Figure 2b) were vertical to those on the second part (shown in Figure 2c). Other information of these features, like dimension, length, and position, were all kept the same in these two parts. The whole design was shown in Figure 2a. Since too many features were printed on the specimen, some were neglected in this work. The square structure was both representative and easy to measure, so they were systematically studied in this work.

The overall performance of the printed specimens were mainly shown using Industrial Computed Tomography (CT), Yxlon FF35 CT, Hamburg, Germany. The X-ray tube used in this research study was FeinFocus® (Stamford, CT, USA) 190 kV nano-focus and selected the QualityScan CT mode. Since the specimen was too thick for laser to penetrate through the side face directly, laser cutting (Bodor S3015, Jinan, China, with laser power 1200W) is also used in this research. Trilinear Coordinates Measuring instrument, Hexagon Metrocogy, RA-7525SEI-4, Sweden and Polyworks (software, 2016) also measured some data to make it more accurate. White light interference profilometry (RTEC MFD-D, San Jose, CA, USA) was also taken to observe the microtopography of the printed parts. A metallurgical microscope (DM2700M, LEICA, Wetzlar, Germany) was used to see the microscopic morphology of the printed parts.

## 3. Results and Discussion

**Figure 3:**
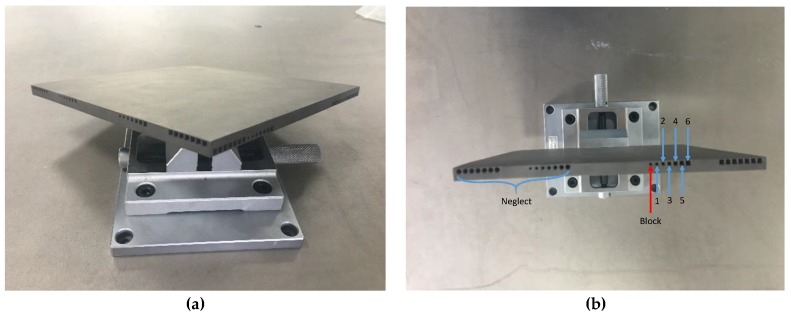
(**a**) The printed specimen and (**b**) the measured position in this work.

**Figure 4:**
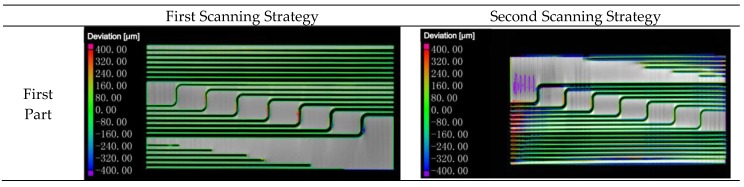

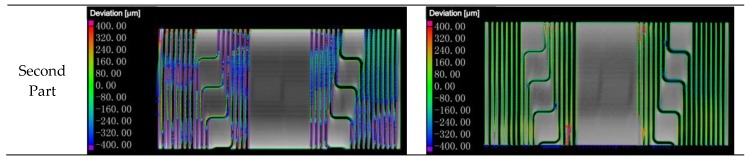
Fitting images of the specimens printed by two scanning strategies. To make the results clearer, every specimen is separated into two parts.

The specimens were built by the first two scanning strategies shown in Figure 2a,b. Since some inner structures in this design sketch were scale animation and some circle features were blocked by the un-melted powder, which were hard to measure the dimension accurately, they were neglected in this research study. The measured positions in this work were shown in Figure 3. The performance of the inner structure on dimension accuracy was measured in industrial CT. It uses the X-ray to scan the specimen directly. When the X-ray passes through the specimen, the intensity will decrease due to the absorption or scattering of Ti6Al4V. Relevant data was collected and a fitting image was made, as shown in Figure 4.

From Figure 4, it was quite clear that the deviation between the first part of the first specimen and the design model ranged from 0 to 80 um, which was almost the same as the deviation between the second part of the second specimen and the design model. The deviation of the other two parts was, however, far worse when compared to these two parts. In another word, the first part printed by the first scanning strategy and the second part printed by the second scanning strategy were better compared to two other parts since the deviation between the design model and manufactured specimen of the inner structure was much smaller. To make an intuition comparison, the exact dimension of the two specimens, which were printed by the first two scanning strategies on the first part, was measured and shown in Table 3. The inner structure with the same design diameter was measured five times on each cross section and each feature selected three cross sections to make the measured diameters more accurate. In addition, to make the trend clearer, the measured data was calculated by using the following equation.

(1) w=|(A−A0)/A0|

W is the deviation between the measured data and design data, A is the measured data, and A0 is the design data.

**Figure 5:**
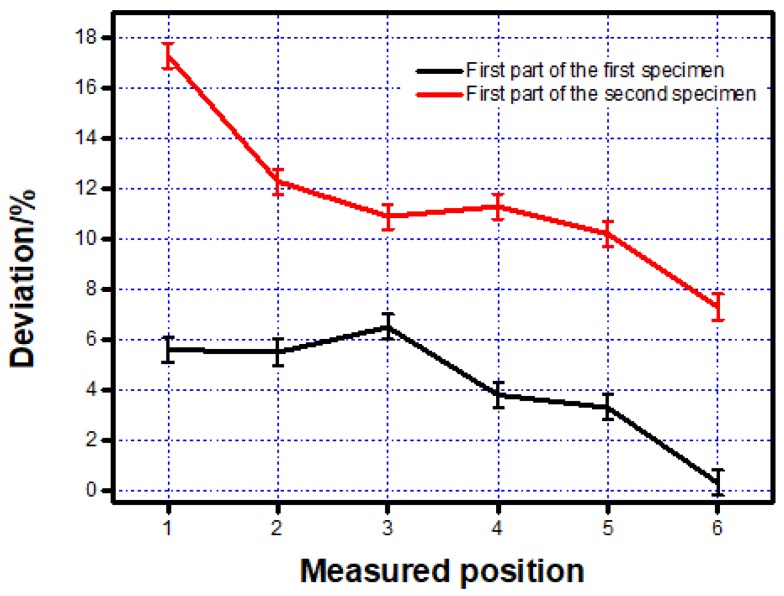
Deviation between the measured and designed data of the two specimens on the first part.

The deviation between the measured data and design data were put in the line charts to make it easier to compare, as shown in Figure 5. The square inner structure with a 1300-µm side length blocked by some powder, which was not melted in some places, was abandoned in this research.

From the line chart shown in Figure 5, it can be found that the deviation of the first part printed by two scanning strategies showed a significant difference. The deviation of the first part printed by the first scanning strategy was at least two times smaller than that printed by the second strategy. 

**Figure 6:**
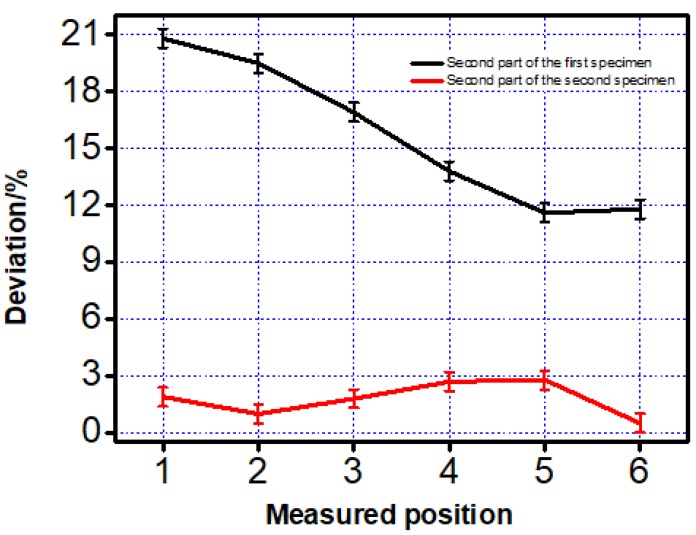
Deviation between the measured and designed data of the two specimens on the second part.

A similar work was also completed on the second part and the dimension of the two specimens was shown in Table 4 and Figure 6.

From Figure 5 and Figure 6 given above, a similar trend was found. Dimension of the features printed along the printing direction was more accurate than those printed vertical to the printing direction. This phenomenon was mainly caused by two reasons. The first reason was when the inner structure did not print along the scanning direction, laser has to on-off frequently, which was difficult to mode locking for the laser and it caused the frequency instability of the laser source [16]. The mode-locking of lasers resulted in the temperature instability on the powder and that was the reason why the inner structure printing along the scanning direction showed a much better property than those printed vertical to the scanning direction. Another reason was the different temperature distribution caused by two different scanning strategies. To illustrate it better, finite element analysis (FEA) was taken to check the temperature distribution after a 0.1-second cooling. Because of the computational workload of ANSYS (Canonsburg, PA, USA), this paper selected the small-size specimen and adopts different grids for the substrate and the specimen to improve computational efficiency. First, the Solid70 thermal element in ANSYS was selected for meshing. The specimen size was 1 mm × 0.6 mm × 0.3 mm, the size of the square hole is 1 mm × 0.2 mm × 0.2 mm, the material of the powder was Ti6Al4V, and the mesh size was 0.05 mm × 0.05 mm × 0.05 mm. The material of the base plate was domestic brand TC4 (Shenzhen, China), and the size was 1.6 mm × 1 mm × 1 mm. The base plate was divided into upper and lower parts. The lower part adopted the larger grid and the mesh size was 0.2 mm to save calculation time. The upper part adopted free mesh to realize the transition of a hexahedron with different sizes.

Since Solid70 supported only one degree of freedom load application, and the powder bed surface convection, radiation and heat flux density of three kinds of load adopted the unit Surf152 surface effect. 

Applying heat flux in the load at the same time, in the powder bed surface building heating unit applied thermal radiation, and to establish a space node powder absorbs heat coming from the bed surface, the conservation of energy was ensured.

**Figure 7:**
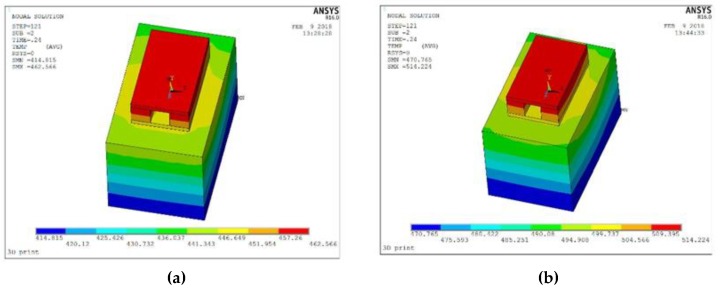
Distribution of the temperature field after scanning. The object on the left uses the scanning strategy printing along the feature (**a**) while the object on the right uses the scanning strategy-printing vertical to the feature (**b**).

The type of heat source used throughout the simulation was a Gaussian heat source, using APDL commands to achieve its loading. First, the temperature was set to room temperature and the steady state initial value analysis was carried out for the whole model. Then, according to the scanning strategy, the printing path was planned. The first scanning strategy vertical to the tunnel started with printing from the bottom left of the model and printing in the positive direction parallel to the x-axis. The second kind of scanning strategy, which was along the tunnel, was consistent with the first scanning strategy and printed along the positive direction parallel to the Z-axis, as shown in Figure 7. Moreover, other process parameters were referenced in Table 2.

**Figure 8:**
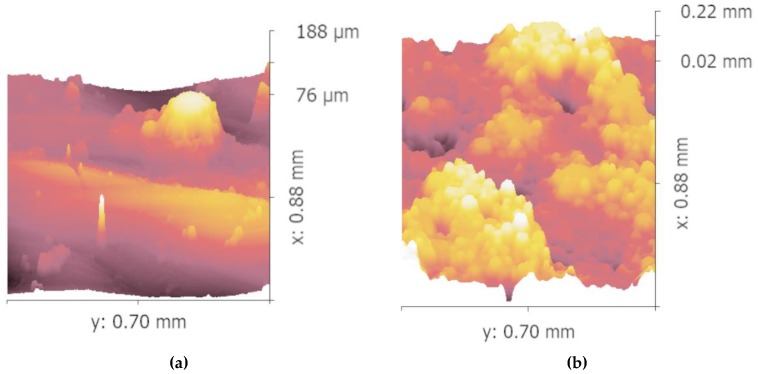

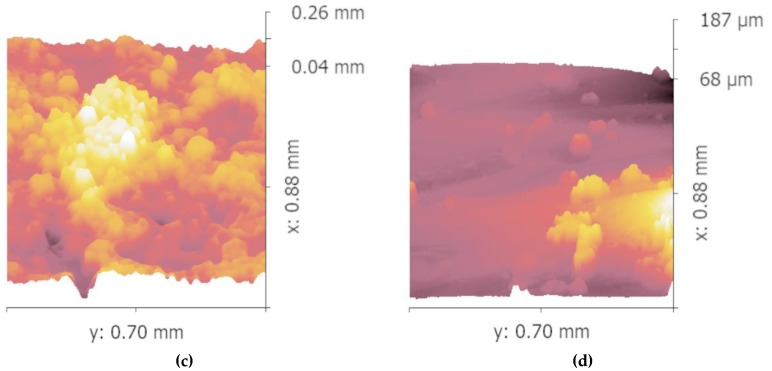
Micro-topography images on the edge of the inner structure of the first part printed by the first scanning strategy (**a**), second part printed by the first scanning strategy (**b**), first part printed by the second scanning strategy (**c**), and second part printed by the second scanning strategy (**d**), respectively.

Figure 7a shows the distribution of the temperature field perpendicular to the Z-scan of the channel after 0.1 s is completed. The maximum temperature is 462.566 K, the minimum temperature is 414.815 K, and the temperature difference is 47.751 K. Figure 7b show the distribution of the temperature field parallel to the Z-scan of the channel after 0.1 s is completed, the maximum temperature is 514.224 K, the minimum temperature is 470.765 K, and the temperature difference is 43.459 K. It can be seen that the temperature difference is smaller after the scanning strategy is parallel to the channel. With a more uniform temperature distribution, surface tension caused by a temperature difference of the molten pool decreases. Marangoni flow also decreases and reduces the stress concentration at the same time [17]. Moreover, the non-uniform temperature distribution leads to the accumulation of the powder, which also results in the dimensional deviation of the inner structure [18]. To further verify these, white light interference profilometry was taken to check the micro-topography on the edge of the inner structure on the side surface, as shown in Figure 8.

**Figure 9:**
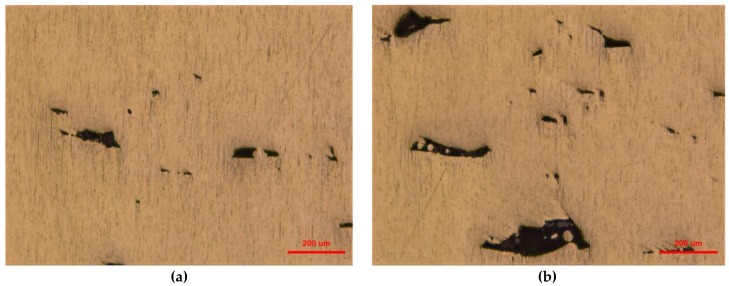

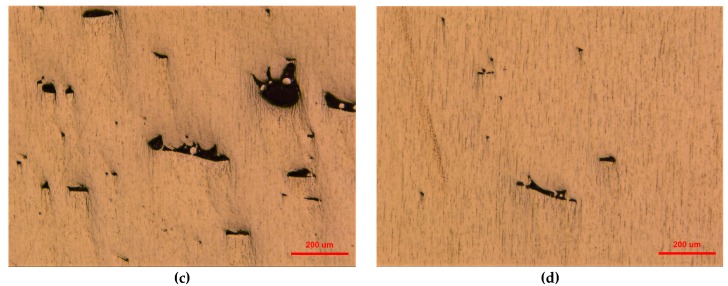
Microscopic morphology of the first part printed by the first scanning strategy (**a**), second part printed by the first scanning strategy (**b**), first part printed by the second scanning strategy (**c**), and second part printed by the second scanning strategy (**d**), respectively.

A significant difference can be seen in these four micro-topography images. Powder accumulation can be clearly seen in Figure 8b,c while the accumulation of the powder is far less serious on the inner structure shown in Figure 8a,d. A similar trend can also be found in Figure 9 as the pits of the first part printed by the first scanning strategy (Figure 9a) and second part printed by the second scanning strategy (Figure 9d) are far less when compared with the pits of the second part printed by the first scanning strategy (Figure 9b) and the first part printed by the second scanning strategy (Figure 9c). This phenomenon conforms to the explanation given above.

Thus, the specimen printed along the inner structure shows a more uniform temperature distribution and ensures that the whole channel printing is more accurate, which will cause a dimensional inaccuracy of the inner structure.

According to the experimental data, the scanning strategy was modified when printing the third specimen. All of the features on two parts were printed along the scanning path, which means the scanning strategy was changed when printing the two parts, as shown in Figure 1c. The accurate layer to change the scanning direction was calculated using the following equation.

n = (0.5H/h) + 1 (2)

n is the number of the layer to change its scanning direction, H is the thickness of the specimen, and h is the layer thickness set in the system.

**Figure 10:**
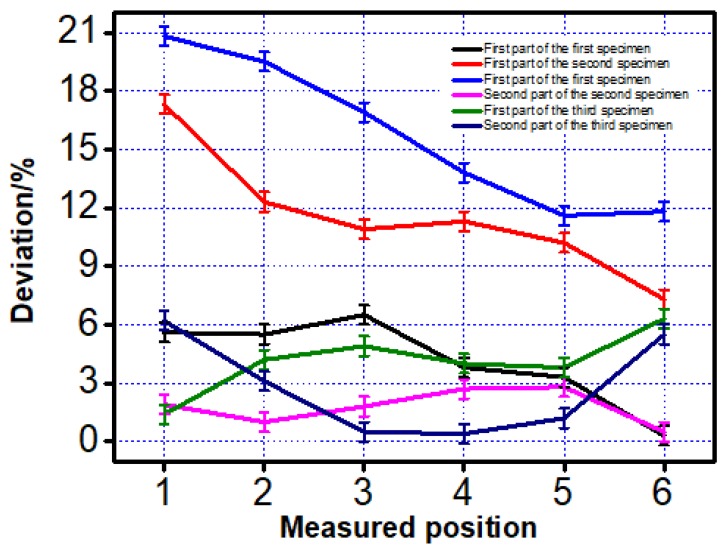
Deviation of the three specimens on both the first and second part.

The measured dimension of the two parts are shown in Table 5. In addition, the comparison of these three specimens was made part-by-part, which means that the three specimens were divided into two parts (the first part and the second part) separately. This makes it easier to compare the dimension accuracy of the printed specimens shown in Figure 10.

From the line chart given above, a conclusion can be drawn that the dimension accuracy of the printed inner structures is modified by using the new scanning strategy.

## 4. Conclusions

In this work, the relationship between the scanning strategy and dimension accuracy of the inner structure is studied and findings are:

(1) The inner structure printed along the scanning direction has a better accuracy than the printed vertical to the scanning direction on dimension accuracy.
(2) The finite element analysis results show that the temperature distribution along the scanning direction is uniform when compared with the printing vertical to the scanning direction, which will cause a dimensional deviation during printing.
(3) Combined with the results given above, a new scanning strategy is proposed and the experimental results show that the dimension accuracy of the inner structure printed by this scanning strategy performs better than those printed by the previous two scanning strategies. The deviation of the dimension between the design model and printed specimen is at least two times smaller when compared with the specimens printed using a one-direction scanning strategy.

Future Work: We will still focus on the quality of the inner structure and optimize accuracy using different methods.

## Figures and Tables

**Table 1:** Relevant information about the powder, which is used to fabricate the specimens.

Powder	Sphericity	Flowability/s	D10	D50	D90	O/%	N/%	H/%	C/%	Al/%	V/%	Fe/%
Ti6Al4V	0.924	26.2	27.7	37.0	49.0	0.175	0.024	0.0064	0.011	5.74	3.83	0.05

**Table 2:** The relevant processing information about the Farsoon FS271M.

Parameter	Laser Power	Laser Scanning Velocity	Layer Thickness	Hatch Spacing	Spot Size	Maximum Printing Size	Protective Gas
Value	180 W	1 m/s	0.05 mm	0.2 mm	100 µm	275 × 275 × 320 mm^3^	Argon

**Table 3:** Measured data on the first part of the two specimens printed by the first two scanning strategies.

		First Specimen		Second Specimen	
Measured Position	Design Sketch (µm)	Average Value on These Three Positions (µm)	Deviation (%)	Average Value on These Three Positions (µm)	Deviation (%)
1	1500.000	1416.227	5.6	1240.653	17.3
2	1700.000	1605.700	5.5	1491.533	12.3
3	1900.000	1776.806	6.5	1692.330	10.9
4	2100.000	2019.448	3.8	1861.871	11.3
5	2300.000	2223.141	3.3	2064.595	10.2
6	2500.000	2492.546	0.3	2318.601	7.3

**Table 4:** Measured data on the second part of the two specimens printed by the first two scanning strategies.

		First Specimen		Second Specimen	
Measured Position	Design Sketch (µm)	Average Value on Three Positions (µm)	Deviation (%)	Average Value on Three Positions (µm)	Deviation (%)
1	1500.000	1187.531	20.8	1471.393	1.9
2	1700.000	1368.053	19.5	1682.852	1.0
3	1900.000	1578.147	16.9	1866.623	1.8
4	2100.000	1810.505	13.8	2043.525	2.7
5	2300.000	2033.572	11.6	2235.255	2.8
6	2500.000	2204.028	11.8	2513.103	0.5

**Table 5:** Measured dimension of the features on both parts of the specimen printed by the modified scanning strategy.

		First Part		Second Part	
Measured Position	Design Sketch (µm)	Average Value on Three Positions (µm)	Deviation (%)	Average Value on Three Positions (µm)	Deviation (%)
1	1500.000	1521.619	1.4	1407.073	6.2
2	1700.000	1770.566	4.2	1647.011	3.1
3	1900.000	1992.769	4.9	1909.767	0.5
4	2100.000	2183.072	4.0	2108.813	0.4
5	2300.000	2387.249	3.8	2326.771	1.2
6	2500.000	2657.917	6.3	2637.273	5.5

## References

[1] Wang D., Yang Y., Liu R., Xiao D., Sun J. (2013). Study on the designing rules and processability of porous structure based on selective laser melting (SLM). J. Mater. Process. Technol..

[2] Yang Z., Yu Y., Wei Y., Huang C. (2017). Crushing behavior of a thin-walled circular tube with internal gradient grooves fabricated by SLM 3D printing. Thin-Walled Struct..

[3] Günther J., Leuders S., Koppa P., Tröster T., Henkel S., Biermann H., Niendorf T. (2018). On the effect of internal channels and surface roughness on the high-cycle fatigue performance of Ti-6Al-4V processed by SLM. Mater. Des..

[4] Seabra M., Azevedo J., Araújo A., Reis L., Pinto E., Alves N., Mortágua J.P. (2016). Selective laser melting (SLM) and topology optimization for lighter aerospace components. Procedia Struct. Integr..

[5] Bremen S., Meiners W., Diatlov A. (2012). Selective laser melting. Laser Tech. J..

[6] Zaeh M.F., Branner G. (2010). Investigations on residual stresses and deformations in selective laser melting. Prod. Eng..

[7] Prem F., Leordean D., Balc N., Pacurar R. The influence of working parameters of SLM technology on surface quality and precision of stainless steel parts. Proceedings of the Annals of MTeM for 2011 & Proceedings of the 10th International MTeM Conference.

[8] Han J., Wu M., Ge Y., Wu J. (2018). Optimizing the structure accuracy by changing the scanning strategy using selective laser melting. Int. J. Adv. Manuf. Technol..

[9] Kruth J.P., Deckers J., Yasa E., Wauthlé R. (2012). Assessing and comparing influencing factors of residual stresses in selective laser melting using a novel analysis method. Proc. Inst. Mech. Eng. B J. Eng. Manuf..

[10] Hodge N.E., Ferencz R.M., Solberg J.M. (2014). Implementation of a Thermomechanical Model for the Simulation of Selective Laser Melting. Comput. Mech..

[11] Li C., Fu C.H., Guo Y.B., Fang F.Z. (2017). A multiscale modeling approach for fast prediction of part distortion in selective laser melting. J. Mater. Process. Technol..

[12] Li C., Liu J.F., Guo Y.B. (2016). Prediction of residual stress and part distortion in selective laser melting. Procedia CIRP.

[13] Zhuang J.-R., Lee Y.-T., Hsieh W.-H., Yang A.-S. (2018). Determination of melt pool dimensions using DOE-FEM and RSM with process window during SLM of Ti6Al4V powder. Opt. Laser Technol..

[14] Vrána R., Červinek O., Maňas P., Koutný D., Paloušek D. (2018). Dynamic loading of lattice structure made by selective laser melting-numerical model with substitution of geometrical imperfections. Materials.

[15] Karg M., Ahuja B., Wiesenmayer S., Kuryntsev S., Schmidt M. (2017). Effects of Process Conditions on the Mechanical Behavior of Aluminium Wrought Alloy EN AW-2219 (AlCu6Mn) Additively Manufactured by Laser Beam Melting in Powder Bed. Micromachines.

[16] Haus H.A. (2000). Mode-locking of lasers. IEEE J. Sel. Top. Quantum Electron..

[17] Hu J., Guo H., Tsai H.L. (2008). Weld pool dynamics and the formation of ripples in 3D gas metal arc wielding. Int. J. Heat Mass Transf..

[18] Dai D., Gu D. (2014). Thermal behavior and densification mechanism during selective laser melting of copper matrix composites: Simulation and experiments. Mater. Des..

